# Genetic interaction mapping with microfluidic-based single cell sequencing

**DOI:** 10.1371/journal.pone.0171302

**Published:** 2017-02-07

**Authors:** John R. Haliburton, Wenjun Shao, Adam Deutschbauer, Adam Arkin, Adam R. Abate

**Affiliations:** 1 Department of Bioengineering and Therapeutic Sciences, University of California, San Francisco, California, United States of America; 2 Department of Bioengineering, University of California Berkeley, Berkeley, California, United States of America; 3 E.O. Lawrence Berkeley National Laboratory, Berkeley, California, United States of America; Tianjin University, CHINA

## Abstract

Genetic interaction mapping is useful for understanding the molecular basis of cellular decision making, but elucidating interactions genome-wide is challenging due to the massive number of gene combinations that must be tested. Here, we demonstrate a simple approach to thoroughly map genetic interactions in bacteria using microfluidic-based single cell sequencing. Using single cell PCR in droplets, we link distinct genetic information into single DNA sequences that can be decoded by next generation sequencing. Our approach is scalable and theoretically enables the pooling of entire interaction libraries to interrogate multiple pairwise genetic interactions in a single culture. The speed, ease, and low-cost of our approach makes genetic interaction mapping viable for routine characterization, allowing the interaction network to be used as a universal read out for a variety of biology experiments, and for the elucidation of interaction networks in non-model organisms.

## Introduction

Cells rely on interactions between biomolecules to achieve complex and dynamic capabilities[1]. For example, cells use genetically encoded signaling proteins to interrogate environmental conditions necessary for adaptation and survival, such as by detecting competitors and responding by secreting an antibiotic. The complete set of biomolecular interactions that a cell uses is often depicted as a connected network known as a genetic interaction diagram[2–4]. With complete knowledge of the interaction network of a cell it is possible, in theory, to predict how the cell will respond to any given stimulus. While achieving such predictive power in practice is not currently possible, even partial understanding of the interaction network is valuable and is a core concept in systems biology[5, 6]. For example, in the study of human health genetic networks are useful for understanding how pathways are dysregulated in disease or drug metabolism. Additionally there is interest in using genetic interactions to better understand novel and synthetic properties of microorganisms, such as the ability to digest environmental contaminants or produce biofuels from cellulosic biomass. Consequently, there is immense interest in novel methods to systematically map genetic interaction networks [7–11].

One way to infer the genetic interaction diagram of a cell is to apply genetic perturbations and observe the impact on a phenotype. By performing two such perturbations simultaneously, it is possible to infer an interaction between a pair of genes [12–14]. For example, if two genes do not interact, the removal of both genes should have a multiplicative effect on phenotype, whereas genes that do interact will produce more complex phenotypes that include suppression or synthetic lethality[15]. The utility and power of a genetic interaction network grows as an increasing number of pairwise interactions are characterized, and is greatest and most detailed by an exhaustive mapping of all possible pairwise interactions[16].

Model systems, like the budding yeast *Saccharomyces cerevisiae* and the bacterium *Escherichia coli*, were some of the first used for systematic genetic interaction mapping, due to the ease with which they can be manipulated[10, 14, 17–21]. This facilitated the development of the single- and double-knockout libraries needed for these studies[22, 23]. However, while generating massive libraries of double knockouts is technically feasible in these microorganisms, screening their phenotypes is far more difficult. For example, screening every pairwise genetic knockout in the *S*. *cerevisiae* genome, comprising ~6,000 genes, requires screening of ~20 million strains. Even with recently-developed high-throughput colony methods, only thousands of combinations can be measured simultaneously, a minute subset of the space of possible combinations[18]. Consequently, to make best use of these screens, much care must be taken in selecting which genes to test as queries; this is not always possible and, even when it is, represents a biased means of mapping the interaction network, since it is only possible to detect interactions that are tested[24].

In this paper, we describe a method for comprehensively mapping genetic interactions. The key to the method is the use of microfluidics to isolate single cells in picoliter droplets at extremely high-throughput. Once confined in the droplets, single-cell linkage PCR physically links the genetic perturbations into a single DNA sequence for analysis by next-generation sequencing (NGS) [24, 25]. This, in essence, converts a library of living cells into a library of DNA molecules, wherein each molecule contains sufficient information to determine genotype of the cell from which it originated. Moreover, since the sequencing depth of a specific sequence is proportional to the relative abundance of the corresponding strain in the culture, the fitness of each strain can be estimated by comparing its membership in the population[26, 27]. This makes our approach supremely scalable: Whereas comprehensive screening of double knockout libraries of yeast or *E*. *coli* would require >10,000 high-density plates, our method can theoretically perform the same screen in a single culture. The speed and ease of our approach will enable the generation of genetic interaction networks in a variety of experimental conditions in diverse microorganisms. For example, rather than just screening a double mutant library in a single conditions (such as rich media), our approach can be adapted to screening multiple conditions, including temperature changes, altering the starting composition of the population, or including chemical perturbations. The availability of conditional genetic interaction networks will be useful for elucidating the cellular logic that underlies environmental sensing and adaptation, and may enable the identification of new drug targets.

## Methods and results

**Fig 1:**
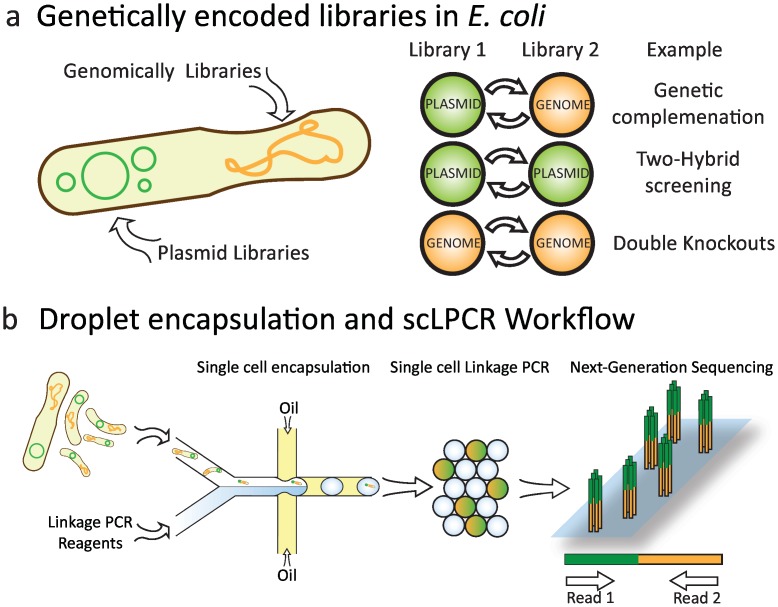
Screening genetic interaction libraries by single-cell sequencing with droplet microfluidics. (a) Genetic libraries can be genomically encoded or introduced through episomal DNA like plasmids. Interaction libraries are created by combining two genetic libraries. Some of the most common types of interaction libraries are noted. (b)Libraries are screen by microfluidic encapsulation and single-cell linkage PCR (scLPCR) inside picoliter droplets. Confining cells inside of droplets allows PCR to link cellular DNA without crossover contamination of DNA from other cells. The PCR products are sequenced using paired-end chemistry on an Illumina platform to decode linkage products.

Mapping genetic interactions requires comparing the phenotypes of single gene perturbations to the phenotypes of double gene perturbations. Making libraries of single genetic knockouts is straightforward, but producing libraries of double mutants is supremely challenging. A common way to produce this library is to cross libraries of single knockouts to generate strains containing defined double-knockout combinations. Alternatively, the single-knockout library can be complemented with a library of additional genes of complementary function (Fig 1a). Genetic interactions within the libraries are scored by measuring the fitness (or growth) of each double mutant strain. Moreover, the culture conditions can be varied, such as by depriving the cells of an important nutrient or adding a drug, to study how genetic interactions change under these conditions. This can be used, for instance, to elucidate the targets of a drug or to deduce key proteins important for signal processing.

A challenge in performing the mapping is tabulating all double knockouts with their fitness under the screening conditions. One way to accomplish this is to isolate each strain at a known location on a plate, and to measure colony growth at that spot. Since the knockout combination at each spot is known, it is straightforward to assign a fitness value to the perturbations. A limitation of this method, however, is that it is onerous to scale at the level needed to completely map genetic interactions in even the simplest cells, due to the need isolate each combination at a spot; this necessitates expensive robotics in addition to immense amounts of reagents and person-hours. Consequently, in most genetic interaction screens, only a small subset of possible interactions is tested. However, deciding which genes to test is not always straightforward and, even when it can be done, the screen will be biased, capable of discovering only interactions that are tested.

An alternative would be to combine all library members into a single, pooled culture, and to quantify population abundance afterwards without having to position each knockout combination on an array. While this is possible with single knockout libraries by “barcoding” strains prior to screening[28], it is not with double knockouts. To barcode strains, a unique identification sequence is associated with each knockout. To quantify population abundance, the barcodes can be amplified with PCR and counted by sequencing[26, 29]. While it is possible to barcode each perturbation separately in a double mutant, it is not currently possible to determine which *combination* of barcodes exists within each cell in a random, high-throughput manner. For example, if a population of double-knockout strains is subjected to PCR to amplify the barcodes, the resultant amplicons for all cells would mix in solution, abolishing information about which pairs existed within the original cells. Retaining this information requires a means for associating together barcode pairs within single cells. Such a method would be very powerful because it would allow a large number of genetic interactions to be screened and retroactively scored in a single, pooled culture.

Our strategy to enable this optimally scalable approach to genetic interaction mapping is to use single cell droplet PCR to fuse barcode combinations into single molecules; these chimeric molecules can be sequenced in massively parallel fashion using NGS (Fig 1b). Moreover, since the sequencing depth of a particular barcode (or barcode pair) is proportional to its abundance in the culture, the fitness of each strain can be estimated by relative membership of its barcode in the sequence data. The key enabling feature of our approach is the ability to perform PCR on millions of single cells using microfluidics, an approach we term single cell linkage PCR (scL-PCR). The principle of scL-PCR is predicated on the ability to rapidly encapsulate single cells inside of microdroplets, where PCR can be used to link cellular DNA without contamination from the DNA of other cells (Fig 1b).

**Fig 2:**
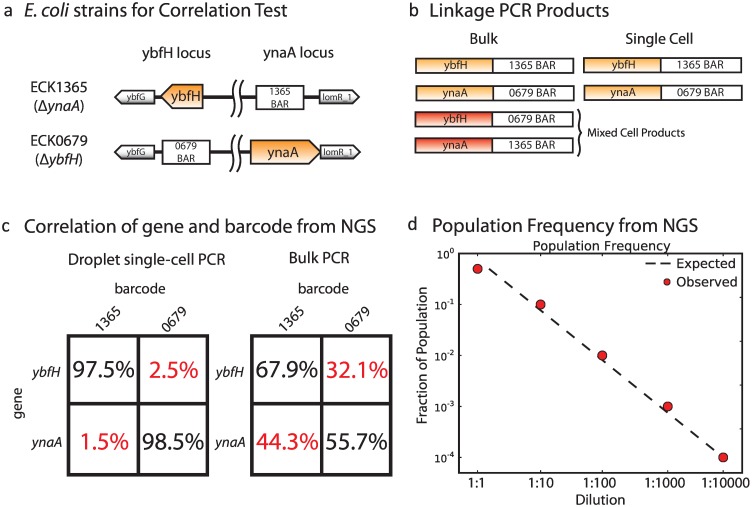
Droplet based single-cell sequencing preserves genomic structure and population membership. (a) KEIO collection strains of *E*. *coli* used to test linkage PCR: a barcode has been inserted into the genome at defined loci, creating gene knockouts of ynaA and ybfH in strains ECK1365 and ECK0679, respectively. (b) Linkage PCR to fuse the sequence from both genomic loci in the two strains yields a mixture of four products in bulk (left), two of which reflect the true genomic organization and two that reflect spurious mixed cell products. However, single-cell linkage PCR (scLPCR) only yields the two products that reflect true genomic organization. (c) Deep sequencing of products from bulk linkage PCR or scLPCR showing percent of reads that reflect true genomic organization (in black) or spurious mixed cell products (in red), indicating the scLPCR on a culture of mixed cell types recovers reports on the genomic variation within the population. (d) The fraction of the population determined by sequencing depth (red dots) when one KEIO strain is spiked into a culture of the other strain at defined dilutions shown on the x-axis. The expected results are shown as a dashed line.

To investigate if scL-PCR faithfully enables the accurate identification of heterogeneous strain combinations in a mixed culture, we prepared two *E*. *coli* strains (Fig 2a, left). Strain ECK1365 contains a knockout at the *ynaA* locus with the 1365 barcode and strain ECK0679 contains a knockout at the *ybfH* locus with the 0679 barcode. The unique barcodes comprise known sequences of 20 bases embedded in a chloramphenicol selection marker. We perform linkage PCR using primers that will link the barcode sequences with each genetic locus. Performing linkage PCR in bulk, as expected, yields chimeric products comprising all four random combinations (two barcodes, two open reading frames); this is because bulk PCR allows the amplicons of both cells to mix in solution, generating chimeric products that consist of sequences from both cells, and which do not represent the genotypes of either cell. By contrast, if the linkage PCR is performed on single cells the only fusions that are generated correspond to the true genotypes of the cells (Fig 2b).

Our method confines this single cell reaction in picoliter droplets using a microfluidic dropmaker (S1 Fig). Because these droplets can be generated at >1 kHz, our approach can process millions of cells per hour; using higher throughput droplet generation techniques, throughputs of billions of cells are achievable. To demonstrate this, we grew the two E. coli KO strains described above separately and pooled them before encapsulation. The cells are individually encapsulated in droplets using microfluidic flow focusing at a concentration limiting dilution such that only 1 in 10 drops contains a cell. For comparison, we also aliquot a portion of the mixed cell population into a PCR tube and perform the LPCR in bulk. The products of the droplet and bulk reactions were prepared for NGS and sequenced using a paired end format, where the sequence from each read reports on a single genetic locus. The droplet workflow yields products accurately reflecting the genotypes of the original populations (Fig 2c, *left*), whereas the bulk reaction shows the expected mixed products (Fig 2c, right). This demonstrates that scLPCR in droplets preserves the genotypes of the strains.

In addition to determining the genotype of each double mutant strain as described above, genetic interaction mapping also requires that we assign a fitness value to each double mutant combination. This can be accomplished by counting the number of instances of each barcode fusion in the sequencing data. As an illustration, prior to encapsulation in droplets we mix the strains at different ratios from 1:1 to 1:10,000 cells. We find that sequencing depth accurately reflects membership library over the four order-of-magnitude range (Fig 2d) that we tested. This demonstrates that read counting is an accurate means by which to quantify strain fitness.

**Fig 3:**
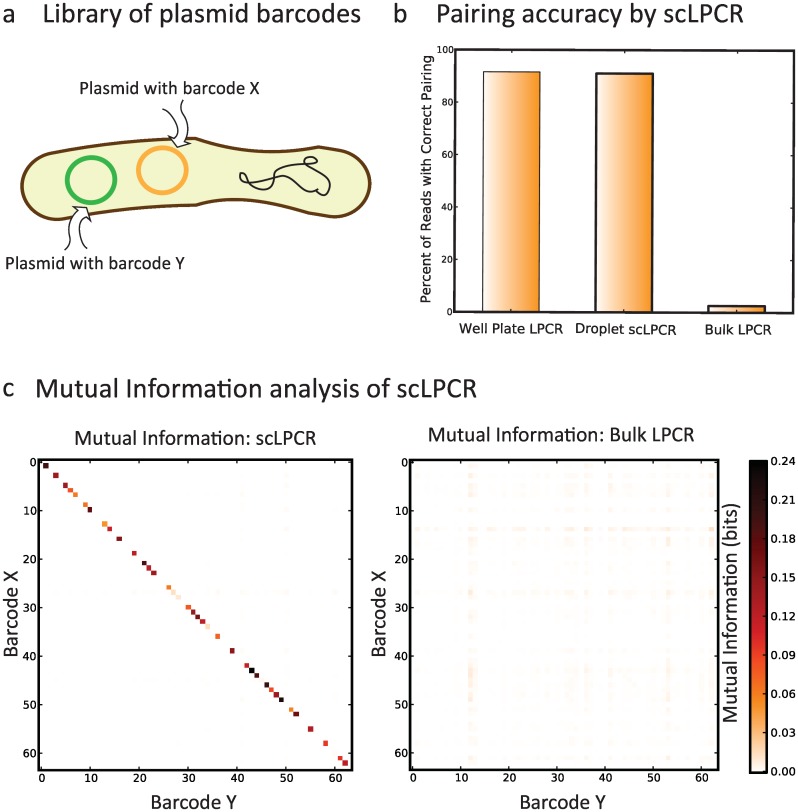
Screening complex libraries with droplet sc-Seq. (a) Library of *E*. *coli* containing 64 strains, each containing a pair of known barcodes (denoted X and Y) located on separate plasmids. (b) The accuracy of barcode X and Y pairing from NGS (as percent of sequencing reads that report a correct X/Y pair) is the same when using linkage PCR on isolated strains (well plate LPCR) or when using single cell linkage PCR in droplets (Droplet scLPCR), while linkage PCR from all strains in bulk (Bulk LPCR) yields mostly random X/Y pairs. (d) The amount of mutual information between specific X/Y barcode pairs in the NGS data shows strong correlations along the diagonal, representing true X/Y pairs. In the same data for libraries from linkage PCR in bulk there is no correlation between represented barcodes.

Genetic interaction mapping can be accomplished by performing gene perturbation combinations that are genome-to-genome or genome-to-plasmid. Alternatively, they can also be performed via plasmid-to-plasmid interactions (for example, with two CRISPR-Cas9 constructs). To conceptually illustrate this, we created a library of 64 *E*. *coli* strains containing unique barcodes encoded on two separate plasmids (Fig 3a). These plasmids were adapted from a two-hybrid strategy for detecting protein-protein interactions in bacteria. The 64 individual strains were grown from frozen glycerol stocks and combined into a single, pooled population. The pool was subjected to the droplet workflow and the resulting scLPCR products were sequenced. As a control, we grew and performed the linkage PCR for the 64 strains individually. The percentage of reads that match the known barcode combinations are the same for the droplet scLPCR method and the individual mapping method, demonstrating that the droplet method performs optimally. In contrast, a bulk reaction control again yields mostly mixed products (Fig 3b). Interestingly, only ~94% of reads for either droplet or isolated strain experiments match the known strain genotypes. We believe this to be due to spurious gene fusions (chimeras) generated during NGS library preparation, which requires a bulk PCR on the mixed products, which may lead to additional fusions. The frequency of these fusions may be reduced by optimizing sequencing preparation and by employing compartmentalized amplification methods, such as emulsion PCR. See the supplementary methods for a more in-depth discussion of noise and experiment design (S1 File).

A valuable means by which to estimate the effectiveness of our method is to plot the mutual information (MI) between the known and measured pairwise gene interactions (Fig 3c). Mutual information is a measure of the confidence with which the presence of one barcode can be associated with that of another. The barcode identities are plotted along the axes and ordered such that correct fusions fall along the diagonal, where the color of the bin is proportion to the MI between the barcodes. For the droplet scLPCR, there is substantial MI between the barcodes on the diagonal, which represent the true sequences of strains in the library. In contrast, the bulk PCR shows little MI for all combinations, which indicates that pairing is random. Peculiarly, there are gaps where known barcode pairs should be present (Fig 3c, *left*). This is likely due to the level of that strain in the population being too low to detect with the sequencing depth that we used. Likely, deeper sequencing would pull out these less-abundant strains.

The speed, ease, and low cost of scLPCR make it valuable for screening the *conditions* under which the cells are cultured, which is useful for investigating how genetic interactions mediate responses to environmental conditions. To illustrate how this can be used to answer a biological question, we generated a new genetic interaction library for amino acid auxotrophy. The library contains 6 strains of *E*. *coli* with single gene deletions, wherein a unique DNA barcode has been inserted into the genome of each strain at that locus. In five of the strains, the deleted gene is essential for amino acid biosynthesis, such that these strains are unable to grow in media not supplemented with the essential amino acid. We also construct four barcoded complementation plasmids that express one of the amino acid biosynthesis genes. If the strain with the deleted gene is complemented with a plasmid encoding that gene, it can synthesize the needed amino acid and, thus, should go in the deficient media. We transformed the set of six strains with the library of four complementation plasmids (24 total strains). The transformed library was recovered for a short time in rich media, washed 3 times in minimal media, and split into two new cultures. One culture was grown in rich media and the other was grown in minimal media. The cultures were grown for 16 generations with periodic dilution to keep them in exponential phase. The cultures were sampled periodically and analyzed by the droplet scLPCR workflow to measure culture membership. We kept the optical density of the cultures low to minimize crosstalk between cells in the culture and to ensure that other media nutrients do not become limiting.

**Fig 4:**
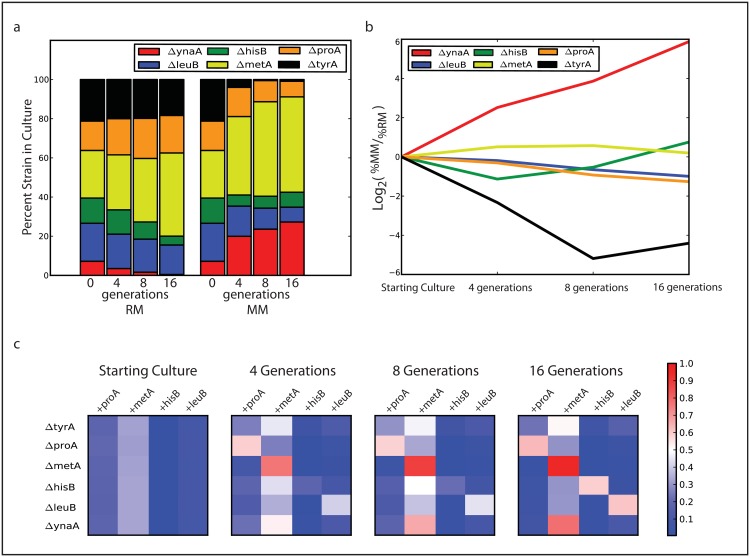
Screening a combinatorial library of amino acid auxotrophy with sc-Seq. (a) The membership (by strain) of heterogeneous cultures is tracked by droplet scLPCR for cultures grown in rich media (RM) or minimal media (MM) at 0,4,8, or 16 doublings after inoculation. (b) The fractional fold change in minimal media vs. rich media over 16 doublings shows that auxotrophic strains with no complement drop out of the population (ΔtyrA, black line) while prototrophic experience no selection and take over (ΔynaA, red line). (c) Droplet scLPCR shows the culture composition by strain and plasmid and unmasks the mechanism of complementation, whereby auxotrophic strains persist in the culture through selective outgrowth of only those strains that harbor the corresponding complementary gene (color corresponds to fraction of sequencing reads within each strain that are specific for the corresponding complement gene).

Using scLPCR, we tracked the culture membership over the 16 generations (S2 Fig), finding that culture composition changes in rich and minimal media (Fig 4a). The proportional difference in composition between rich and minimal media at each time point reflects the biological impact of amino acid auxotrophy (Fig 4b). The *ynaA* knockout, which contains no amino acid auxotrophy, should grow equally well in rich or minimal media. As expected this strain is significantly enriched in the minimal media culture. Conversely, the *tyrA* knockout cannot grow in minimal media and cannot be complemented by any of the plasmids in our library; therefore this strain drops out of the culture grown in minimal media. In addition to tracking the membership of the culture by strain, we can track the membership of plasmids within each strain. We find that there is no enrichment for the knockouts of *hisB*, *leuB*, *metA*, and *proA* at the strain level (Fig 4b), but within each strain there is enrichment for cells harboring the needed complementation plasmid (Fig 4c) across the 16 generations of growth. Peculiarly, we also found that cells with the *metA* complementation plasmid persisted in the culture. This observation turns out to be consistent with recent findings suggesting that overexpression of the MetA protein can drive cells towards a persistor phenotype.[30]

## Conclusions

We have demonstrated a method to rapidly screen genetic interactions in a single culture. We produced genetic interaction libraries comprising two genetic perturbations and used single cell linkage PCR and NGS to reliably quantify the levels of every member in the library. This should make our approach useful for non-model bacterial systems, wherein genomic modification (by transposons, CRISPR-Cas9, or targeted modification) is the only requirement. In addition, the massive scalability afforded by droplet microfluidics should enable higher order interactions, such as 3-gene interactions, to be tested.

A key advantage of our approach is the speed and ease with which libraries can be screened across multiple conditions. This allows our approach to be adapted to multiple library types, including genetic knockouts, the addition of biosynthetic pathways and non-native genes, and protein interactions like the classic two-hybrid screen. We envision that this method can be extended to eukaryotic systems for use in medical research and drug development. The elucidation of genetic interaction networks in model systems like *S*. *cerevisiae* and *E*. *coli* capitalized on decades of development in microbiology and precise molecular tools. Though library creation is time consuming, the pictures that emerge are rich in information and provide key insights into genomic design principles, and these libraries continue to be screened and mined for information. The arrival of new molecular tools like the Cas9 system allow these same concepts to be extended to new organisms with ease and it is expected that the library creation process will no longer be rate limiting. The use of droplet microfluidics to deconvolute complex cell libraries is a powerful tool that can be combined with next-generation methods of library creation to allow for truly rapid interaction profiling in a multitude of conditions, time points, and formats.

## Supporting information

S1 File Supporting information. (PDF)

S1 Fig Microfluidic device for sc-LPCR. The microfluidic droplet maker is a co-flow device consisting of a single outlet 3 inlets, one for oil and one each for cells and PCR mix. Aqueous mixes are flowed into a single channel that intersects a perpendicular channel of oil. Drops are made the junction and their size is function of the device geometry at the junction. This device has a width of 25 microns at the dropmaking junction and a height of 25um, which produces drops of approximately 30 microns in diameter.
(TIF)

S2 Fig Growth of strains in auxotrophy experiment. (a) Library of complementation strains grown in EZ-Rich media for 16 generation. Each time the culture reaches O.D. ~0.32 (4 generations) it diluted back to O.D. 0.02. There is an initial lag of culture growth as the strains recover from transformation (Plus 1), but the culture quickly achieves uniform growth rate. (b) Library of complementation strains grown in EX-Min media for 16 generations. For this culture condition the lag phase is very long (Min 1), and each successive culture grows slightly faster.
(TIF)
