# Supplementary material for: Genetic interaction mapping with microfluidic-based single cell sequencing
Source: PLoS One. 2017 Feb 7;12(2):e0171302. doi: 10.1371/journal.pone.0171302 (PMC5295688; doi:10.1371/journal.pone.0171302)
Supplement: S1 File — (PDF) [file pone.0171302.s001.pdf]

Supplementary Information

Methods and Materials:

Fabrication of microfluidic dropmakers

The microfluidic devices are fabricated using soft lithography[Basic Microfluidic and Soft Lithographic Techniques]. SU-8 photoresist (MicroChem Corp) is spun onto a 3”silicon wafer (University Wafer) to a desired thickness and baked at 135°C to remove solvent. A photo transparency mask (CAD/Art Services) containing the device features is placed on the wafer and exposed to UV light to crosslink the photoresist. Following UV exposure, the wafer is post-baked at 135°C for 1 minute and placed into a developing bath of propylene glycol methyl ether acetate (PGMEA, Sigma). Following development with PGMEA, the masters are washed with isopropanol and post-baked at 135°C for 30 minutes. The masters are placed into plastic petri dishes and covered with degassed poly(dimethylsiloxane) (PDMS) prepared from 10:1 ratio of elastomer:crosslinker (Sylgard 184, Dow Corning). The dish is evacuated to remove entrapped air bubbles and baked at 65°C for at least 2 hours to crosslink the PDMS. The PDMS devices are cut with a scalpel and peeled away from the master. Holes for inlets and outlets are punched using a biopsy core (Harris Uni-Core), the devices are rinsed with isopropanol, and they are plasma-bonded to glass slides. The devices are flushed with Aquapel to render the channels hydrophobic and enable water-in-oil emulsification, and baked at 65°C for 20 min to remove excess Aquapel. To operate the microfluidic devices, Polyethylene (PE) tubing (Scientific Commodities) is used to connect device inlets to syringes containing reagents, and a custom Python script used to control syringe pumps and inject liquids into the device. The oil used is Novec HFE 7500 (3M) containing 2% fluorosurfactant (RAN Biotechnologies) and droplets are collected into PCR tubes. Prior to subjecting droplets to thermal treatments, the HFE oil is removed from beneath the droplets with a pipette fitted with a gel loading tip and an equal volume of FC-40 oil (Sigma) containing 5% surfactant is added above the emulsion.

Strains used in this study

Unless otherwise noted, all knockout strains in this study were taken from the ASKA knockout collection [1].

Culture conditions for two-strain experiment

Strains ECK1365 and ECK0679 were separately inoculated in to LB Broth containing 30ug/mL chloramphenicol. Strains were grown to saturation overnight and then used to inoculate fresh cultures at an O.D. ~ 0.005. Cultures were allowed to grow to mid-log (O.D. 0.2) and then pooled at various ratios (1:1, 1:10, etc). Pooled cultures were diluted to an O.D. of 0.005 in ddH2O (~1 cell per 200pL).

Diluted cells were encapsulated with PCR Mix (Phusion polymerase, detergent free buffer) in a co-flow microfluidic device. Devices are 30um in height and use a dropmaking nozzle that is 30um wide, resulting in drops that are ~35um in diameter. Flow rates for each aqueous inlet are 200ul/hr and flow rate for the oil is 800ul/hr.

For bulk experiments, diluted cells are combined with LPCR mix directly in a PCR tube.

Culture conditions for 64 strain experiment

Freezer stocks of 64 strains were inoculated into a deep-well 96-well plate containing 200uL of LB broth 30ug/mL chloramphenicol and 50ug/mL kanamycin. Strains were allowed to grow at $37^{o}C$ for 3 hours. For the well plate control, each well was sampled individually for LPCR. For the droplet experiments strain were combined and diluted to an O.D. of 0.005 in ddH2O (~1 cell per 200pL).

Diluted cells were encapsulated with PCR Mix (Phusion polymerase, detergent free buffer) in a co-flow microfluidic device. Devices are 30um in height and use a dropmaking nozzle that is 30um wide, resulting in drops that are ~35um in diameter. Flow rates for each aqueous inlet are 200ul/hr and flow rate for the oil is 800ul/hr.

For bulk experiments, diluted cells are combined with LPCR mix directly in a PCR tube.

Construction of barcoded complementation plasmids

Barcodes were introduced into plasmid pBbA2k-RFP(gift from Jay Keasling (Addgene plasmid # 35327)) by overlap PCR with primers the contained a 7bp barcode[2]. The plasmid contains a constitutive Tet promoter driving expression of RFP. Each amino acid biosynthesis gene was amplified from genomic DNA and cloned into the plasmid to replace the RFP gene. Cloning was done using Clontech's In-Fusion kit.

Culture conditions for complementation assay

Each ASKA knockout strain (6 total) was made competent and transformed with the set of 4 complementation plasmids. Cultures were pooled and recovered for 3 hours in rich media. Cultures were washed 3 times with minimal media before being inoculated into 50mL of either EZ-Rich Media (Teknova) or EZ-Min Media (EZ-Rich without Amino Acid supplement) at an initial O.D. of 0.02. Cultures were grown at 37°C until the O.D. reached ~0.32 (4 generations), at which point culture were sampled and diluted back to O.D. 0.02.

Sequencing on the MiSeq NGS Platform:

The products of each LPCR reaction were subjected to an additional bulk PCR in order to add sequencing adapters. Products from this second PCR were column purified (Zymo Research) and sequenced on a MiSeq platform using a paired end format and 200bp reads. Reads were analyzed with custom scripts that extracted barcode or gene signals from each read.

Experimental design for measuring genetic interactions by deep sequencing

Traditional methods of measuring genetic interactions use growth on solid agar plate to calculate a fitness value (W) for a particular strain, defined as the area of the colony at some time (t_2) when imaged by a camera[3]. Precise control over the initial seeding density at the beginning of the experiment (t_1) and spatial separation of strains eliminate significant sources of noise.

In liquid cultures of mixed strains the fitness value of a strain is conceptually similar and defined as the fold expansion of each strain relative to the rest of population and is mathematically expressed for strain *i* as:

$$Wi = \frac{\ln\,(N_i(t_2)/d(\,N_i(t_1))}{\ln\big((1-N_i(t_2)\big) * d(1-N_i(t_1)))} \qquad (1)$$

Where $N_i\,(t_1)$ and $N_i(t_2)$ are the frequency of strain *i* in the population at time points t_1 and t_2 and *d* is the ratio of the optical densities at timepoints t_1 and t_2 and represents the growth of the culture[4] Others have shown that deep sequencing can be used on barcoded strains to obtain values for $N_i\,(t_1)$ and $N_i(t_2)$ by using sequence depth as a proxy for $N_i\,(t_1)$ and $N_i(t_2)$. Our method extends this approach to strains with two, and possibly more, barcodes by using single-cell linkage PCR to associate multiple barcodes from cells prior to sequencing. Our results are similar to previous results, as we show that sequencing accurately reflects culture composition across multiple orders of magnitude. When using this method it is important to consider certain parameters and possible sources of experimental noise and how they could convolute results.

Important parameters:

1. Library diversity and Sequencing depth: The confidence of fitness scores will grow as the culture is sequenced deeper. For larger libraries composed of several strains this will necessitate more sequencing. Importantly, because the method is a single-cell approach, it will also necessitate encapsulating more cells. Ideally the strain composition at the start of the experiment will be roughly equal, but for complex cultures that are made from hundreds of freezer stocks the distribution might be uneven and certain strains could drop out if not enough cells are screened and sequenced to capture them. As a general rule, at least 10 times as many cells as there are strains in the library should be screened, and at least 10 times as many reads as cells should be sequenced.
2. Controlling for dropouts: To control for the impact of dropouts, which could lead to false synthetic sick phenotypes, the culture should always be sequenced at the start of the experiment. From this sequencing data, all strains that are not detected with enough reads should be excluded from the experiment. Fit the read depth for all strains to a normal distribution and exclude those strains that don't meet a z-score threshold of at least 2 (~95% confidence).
3. Use of control strains: it can be helpful to include strains with known growth phenotypes, such as those that are known to be synthetic sick, synthetic lethal, or have no interaction. Using the strains will allow regions of the fitness spectrum to be assigned to these phenotype nomenclatures.

Possible sources of noise:

1. Multiple encapsulations: if the cells are seeded into the dropmaker at too high concentration then chimeric barcodes could be created. The impact of these products on the quality of the data should be minimal. In extreme cases there will be so many cells in each drop that the data will look like the mixed-culture control shown in Figure 3, where the frequency of every barcode pair is essentially the same,

this level of contamination will be obvious to the user. Additionally, the use of control strains with known barcodes will enable the user to determine the amount of multiple encapsulations by observing the number of reads with spurious linkages between these barcodes and others.

2. PCR bias in drops: It is also possible that some strains will not amplify well in droplets. If that's the case these strains will fail to pass the dropout filter and will not be included for analysis
3. PCR bias in library preparation: It is known that bias is introduced into sequencing libraries through PCR. We observed a PCR cycle number dependent noise factor in our experiments and found that using a limited number of PCR cycles enabled us to produce libraries with high confidence. However, this source of bias could potentially create chimeric barcodes. Conditions should be optimized to use the bare minimum of PCR cycles needed to prepare sequencing libraries.
4. Culture density: Mixed cultures can influence each other through production of secondary metabolites, which could mask or exacerbate the effects of genetic interactions. It is crucial that the culture density be kept low enough to preclude the build-up of secondary metabolites. For that reason, the culture should be either diluted continuously or diluted when it reaches a certain O.D. threshold of early exponential phase.

References

1. Kitagawa M, Ara T, Arifuzzaman M, Ioka-Nakamichi T, Inamoto E, Toyonaga H, et al. Complete set of ORF clones of Escherichia coli ASKA library (a complete set of E. coli K-12 ORF archive): unique resources for biological research. DNA Res. 2005;12(5):291-9. doi: 10.1093/dnares/dsi012. PubMed PMID: 16769691.

2. Lee TS, Krupa RA, Zhang F, Hajimorad M, Holtz WJ, Prasad N, et al. BglBrick vectors and datasheets: A synthetic biology platform for gene expression. J Biol Eng. 2011;5:12. doi: 10.1186/1754-1611-5-12. PubMed PMID: 21933410; PubMed Central PMCID: PMCPMC3189095.

3. Tong AH, Lesage G, Bader GD, Ding H, Xu H, Xin X, et al. Global mapping of the yeast genetic interaction network. Science. 2004;303(5659):808-13. doi: 10.1126/science.1091317. PubMed PMID: 14764870.

4. van Opijnen T, Bodi KL, Camilli A. Tn-seq: high-throughput parallel sequencing for fitness and genetic interaction studies in microorganisms. Nat Methods. 2009;6(10):767-72. doi: 10.1038/nmeth.1377. PubMed PMID: 19767758; PubMed Central PMCID: PMCPMC2957483.
