# Supplementary figures and images for: Genetic interaction mapping with microfluidic-based single cell sequencing
Source: PLoS One. 2017 Feb 7;12(2):e0171302. doi: 10.1371/journal.pone.0171302 (PMC5295688; doi:10.1371/journal.pone.0171302)

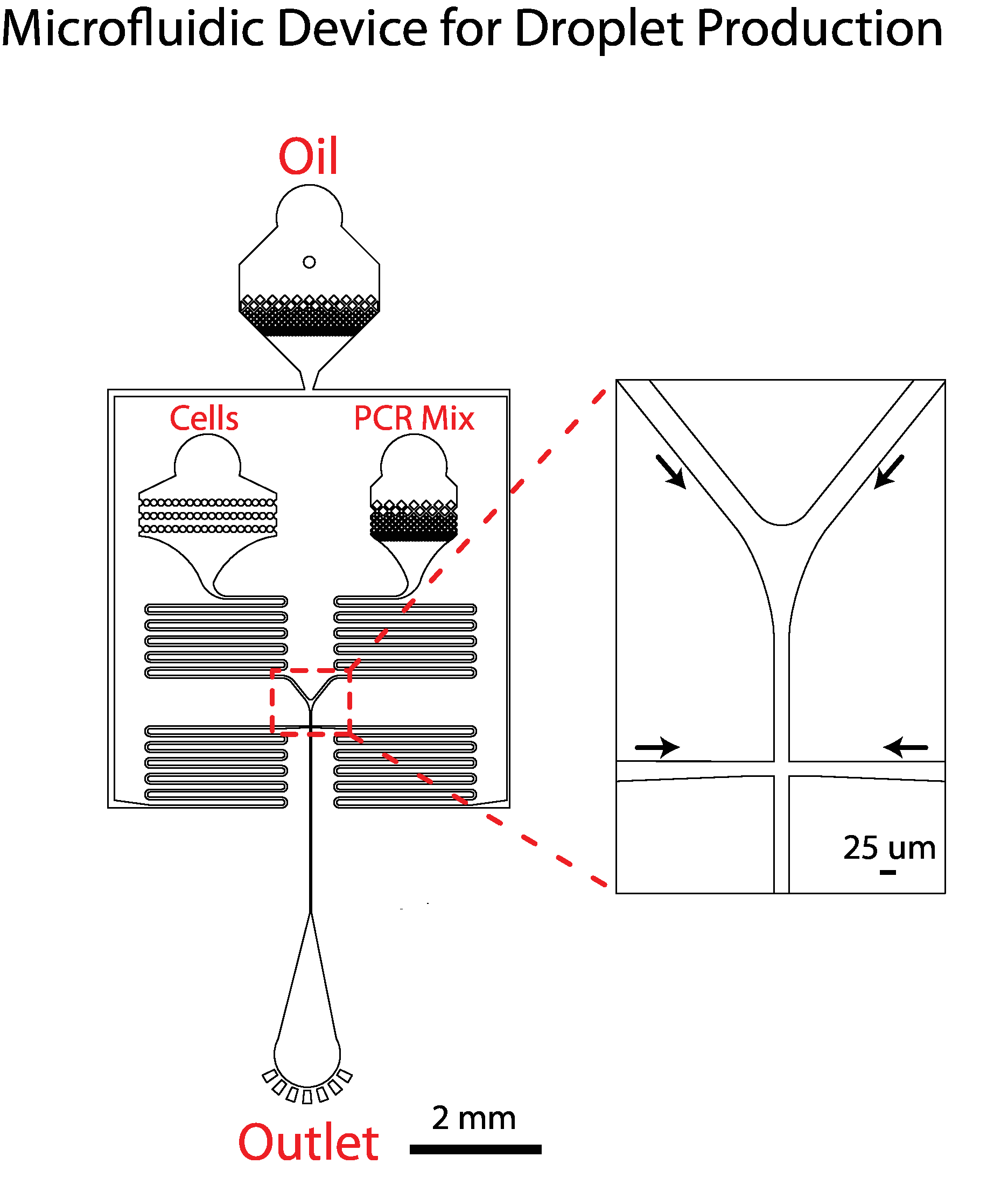

Supplement: S1 Fig — The microfluidic droplet maker is a co-flow device consisting of a single outlet 3 inlets, one for oil and one each for cells and PCR mix. Aqueous mixes are flowed into a single channel that intersects a perpendicular channel of oil. Drops are made the junction and their size is function of the device geometry at the junction. This device has a width of 25 microns at the dropmaking junction and a height of 25um, which produces drops of approximately 30 microns in diameter. (TIF) [file pone.0171302.s002.tif]

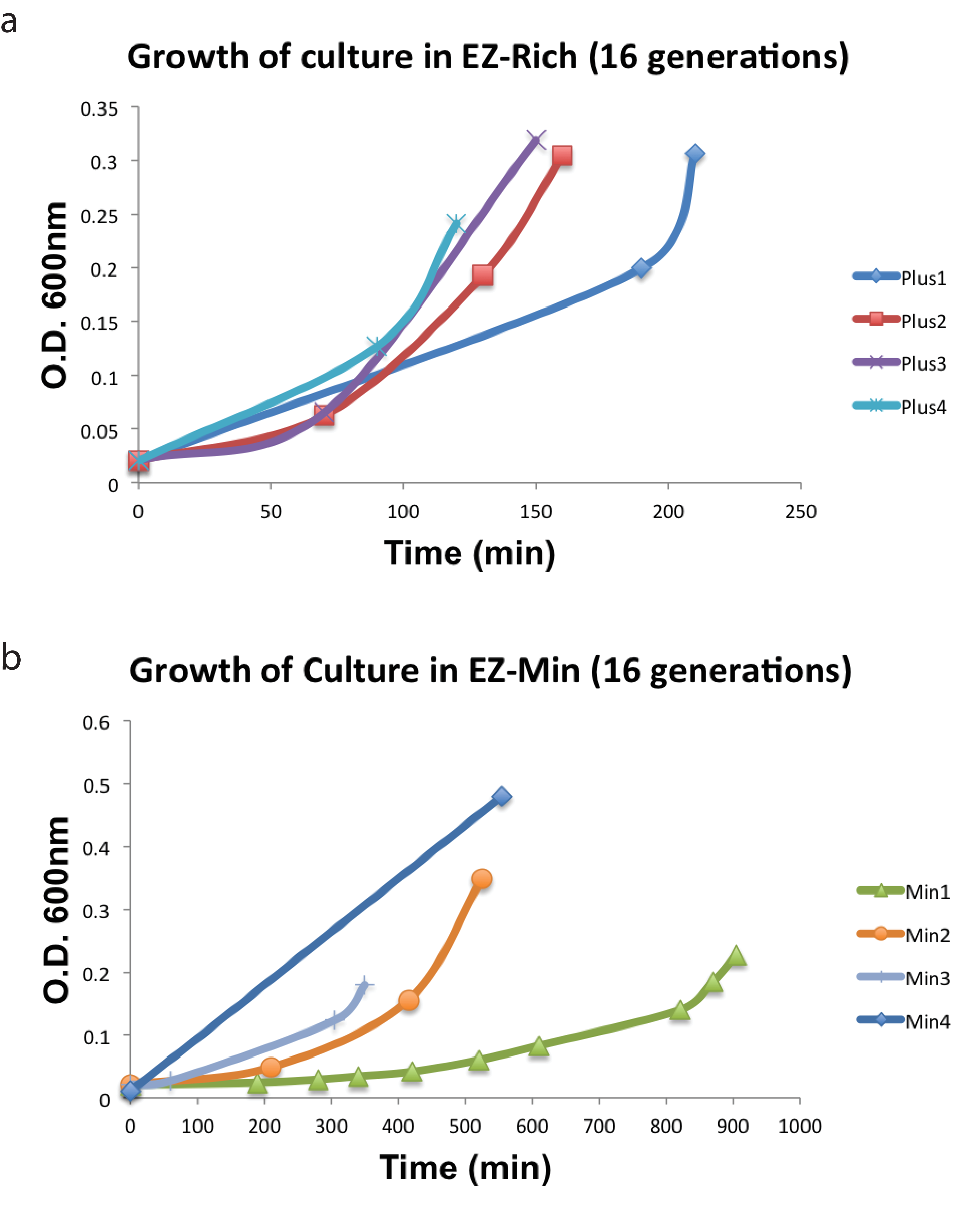

Supplement: S2 Fig — (a) Library of complementation strains grown in EZ-Rich media for 16 generation. Each time the culture reaches O.D. ~0.32 (4 generations) it diluted back to O.D. 0.02. There is an initial lag of culture growth as the strains recover from transformation (Plus 1), but the culture quickly achieves uniform growth rate. (b) Library of complementation strains grown in EX-Min media for 16 generations. For this culture condition the lag phase is very long (Min 1), and each successive culture grows slightly faster. (TIF) [file pone.0171302.s003.tif]
